# Conjunctival Tarsal Actinic Keratosis Treated with Interferon Alfa-2b: A Rare Case Report and Literature Review

**DOI:** 10.1155/2021/6616021

**Published:** 2021-01-22

**Authors:** Mónica Gimeno-Carrero, María-Jesús Suárez-Fernández, Beatriz Alonso-Martín, Almudena de-Pablo-Cabrera, María-Concepción Garrido-Ruíz, Enrique Mencía-Gutiérrez

**Affiliations:** ^1^Ophthalmology Department, 12 de Octubre Hospital, Complutense University, 28041 Madrid, Spain; ^2^Pathology Department, 12 de Octubre Hospital, Complutense University, 28041 Madrid, Spain

## Abstract

Conjunctival neoplasia is one of the most frequent tumors in the eye. Actinic keratosis (AK) or solar keratosis is a precancerous lesion that is included with other epithelial tumors. This alteration does not break the basal membrane. There is enough evidence of successful outcomes to consider interferon alfa-2b (IFN alfa-2b) as the first choice of treatment for this type of tumors. In addition, side effects are mild and uncommon. We report a case in an 83-year-old woman who was referred to evaluate a leukoplakia in the tarsal conjunctiva of the lower left eyelid that measured 1 cm in diameter. Pathological study revealed AK. After the INF alfa-2b treatment, we observed conjunctival hyperemia, noninfiltrated upper nasal de-epithelization, and inferior nasal bulla. AK with presentation in conjunctiva is rarely described and in tarsal conjunctiva is exceptional. It is the first case published with only tarsal conjunctiva affectation.

## 1. Introduction

Actinic keratosis (AK) or solar keratosis is a hyperkeratotic plaque-like lesion (leukoplakia) with defined borders and squamous surface (it is a premalignant lesion because it includes dysplasia keratinocytes). It is frequent in the elderly population and in the light skin since its main risk factor is sun exposure [1]. Due to that fact the presentation in the tarsal conjunctiva is very unusual [2], the tarsal conjunctiva is not directly exposed to ultra violet (UV) radiation, the usual etiological cause of its development. Yearly AK to squamous cell carcinoma progression rates of 0-6% have been reported [3].

Individuals with Fitzpatrick type I or II (I-VI) skin characteristics, such as fair skin, freckles, light-colored eyes (blue or green), and blonde or red hair, are more likely to developed AKs, as they are more sensitive to damage from chronic sun exposure [4].

Interferons are a set of glycoproteins identified in 1957 and produced naturally by the body for their antiviral, antiproliferative, and immunomodulatory properties [5]. To achieve tumor remission, surgical treatment with wide free margins has been performed classically, followed by cryotherapy at the base of the lesion [6]. However, the recurrence rate is high because of the difficulty of achieving noninfiltrated margins.

In addition, the surgical procedure involves a higher cost and causes greater stress and trauma to the patient [5]. Topical treatment with IFN alfa-2b has been shown to have excellent results, so the use of topical treatments like INF alfa-2b is becoming widespread.

The use of topical INF alfa-2b is considered safe, effective, and generally well tolerated. However, side effects due to its systemic absorption have been found. These include flu-like symptoms, migraine or fatigue, and others due to its topical use such as follicular conjunctivitis, conjunctival injection, or superficial punctate keratitis [6–8]. All of them usually remit spontaneously within one month after treatment completion. None of the aforementioned adverse effects constitutes a sufficient reason to discontinue treatment. Few cases affecting the conjunctiva have been published [9], one only with conjunctival eyelid margin involvement [2] and none localized on the tarsal conjunctiva.

## 2. Case Presentation

**Figure 1:**
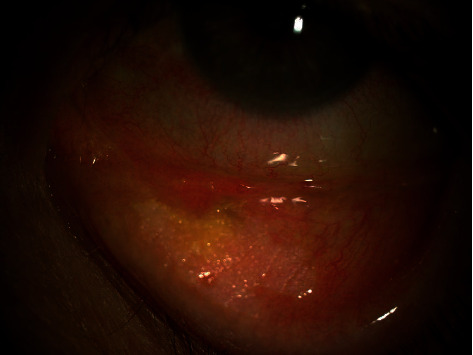
Image with the presence of a leukoplakia measuring 1 cm in length on the lower left eyelid tarsal conjunctiva.

**Figure 2:**
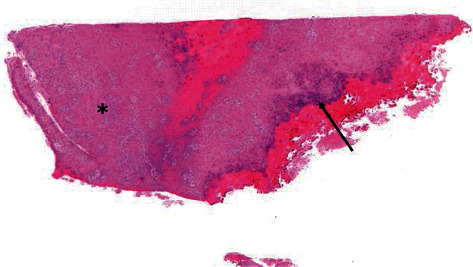
Conjunctival tarsal biopsy fragment showing acanthosis with epidermal hyperplasia (asterisk) and slight papillomatosis and hypergranulosis in superficial layers (arrow). Hematoxylin and Eosin, 4x magnification.

**Figure 3:**
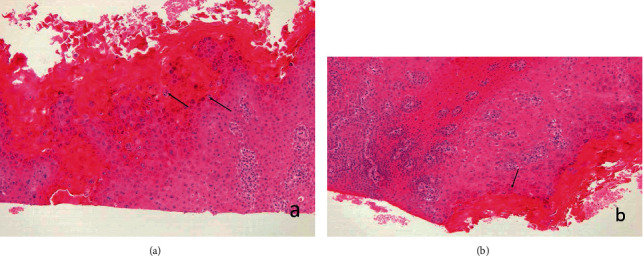
(a, b) Keratinocyte atypia is observed focally (arrows). Hematoxylin and Eosin, 10x magnification.

**Figure 4:**
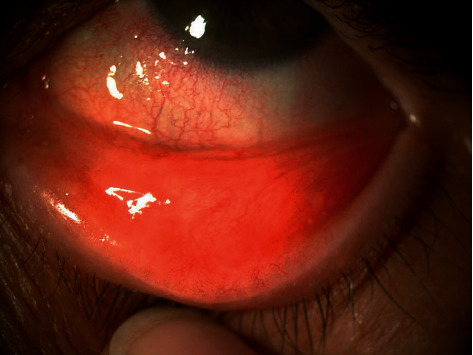
One month after the start of the treatment, the lesion has disappeared in the left eye.

**Figure 5:**
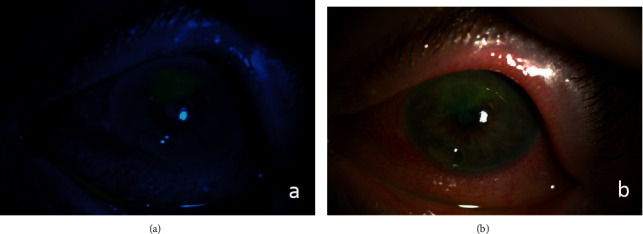
Slit-lamp image of the left eye with conjunctival hyperemia, noninfiltrated upper nasal de-epithelization, and inferior nasal bulla. (a) Image with fluorescein staining and blue filter and (b) image with white light.

**Figure 6:**
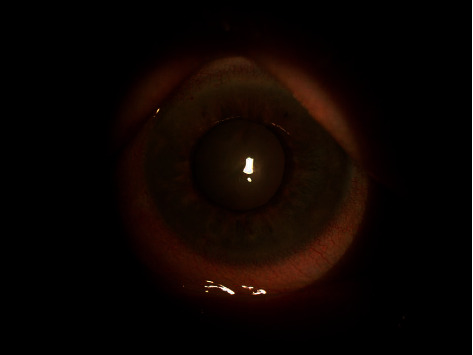
Slit-lamp image of the left eye five days after the cessation of alfa-2b interferon therapy with almost complete disappearance of the signs.

An 83-year-old female referred from the outpatient clinic for a leukoplakia measuring 1 cm in its wide diameter on the tarsal conjunctiva of the lower eyelid of the left eye (Figure 1). The patient had not had prolonged exposure to the sun during her life. She had no history of skin cancer. She was not immunosuppressed. An incisional biopsy of the lesion was sent to the pathology department. The results showed acanthosis with epidermal hyperplasia and slight papillomatosis and hypergranulosis in superficial layers (Figure 2). Focal keratinocyte atypia was observed (Figures 3(a) and 3(b)). A diagnosis of presumed conjunctival AK was made. Treatment with INF alfa-2b one million IU/ml eye drops every 6 hours was started. After one month, the lesion had practically disappeared entirely, so IFN alfa-2b treatment was changed to 1 drop every 12 hours for 15 days and 1 drop every 24 hours for another 15 days (Figure 4). However, the patient came to the emergency room 15 days later presenting conjunctival hyperemia, noninfiltrated upper nasal circular de-epithelization, and inferior nasal bulla (Figures 5(a) and 5(b)); thus, discontinuation of interferon was decided. After 5 days without the INF alfa-2b treatment, the clinical picture was almost resolved, with complete disappearance of the conjunctival hyperemia, near-complete superior nasal reepithelization, and marked reduction of the inferior nasal bulla, with complete disappearance of the symptoms after one month (Figure 6). Follow-up after 18 months showed no recurrence of the lesion or the adverse event that had occurred.

## 3. Discussion

AK (premalignant lesion) is described as neoplastic transformation and proliferation of keratinocytes within the epidermis that does no breach the basal membrane [1]. The histopathology of the keratotic plaque shows acanthosis of the epithelium and keratinization of the conjunctival epithelium and parakeratosis. The AK shows a similar histopathologic aspect with prominent keratosis and usually appears as a chronic inflammation. It must be treated due to the risk of developing SCC. Since they represent the initial stage in the evolution of SCC, recognition and treatment are important. Clinically conjunctival AK and intraepithelial neoplasia (IEN) are indistinguishable. Histopatologically conjunctival AK occurs as a localized, minimally aggressive lesion confined to the surface epithelium. Conjunctival IEN is characterized by a partial-thickness replacement of the surface epithelium by anaplastic epithelial cells that lack normal maturation [1]. They account for less than 1% of conjunctival lesions [1]. Although the most important cause is direct UV radiation exposure, other risk factors have been described: fair skin, advanced age, exposure to carcinogens, immunosuppression, and genetic conditions [10].

To achieve tumor remission, surgical treatment with wide free margins has been performed classically, followed by cryotherapy at the base of the lesion [6]. However, because of the difficulty of achieving noninfiltrated margins, the recurrence rate was high. In addition, the surgical procedure involves a higher cost and causes greater stress and trauma to the patient [5]. In this case, due to the location and size of the lesion, surgical treatment would have required a long and complex procedure with high risk of complications and several follow-up visits, and the result would probably have been nonesthetic, with a worse quality of life. Chemotherapy with INF alfa-2b eye drop treatment was chosen due to its proven effectiveness and despite being an expensive treatment [9]. Topical treatment with IFN alfa-2b has been shown to have excellent results, equal to or greater than those found with surgical excision. Because of these factors, the use of topical treatments like INF alfa-2b is becoming widespread.

The use of topical INF alfa-2b is considered safe, effective and generally well-tolerated. However, side effects due to its systemic absorption have been found; these include flu-like symptoms, migraine and fatigue; other side effects are due to its topical use, and include follicular conjunctivitis, conjunctival injection or superficial punctate keratitis [6–8]. All of them usually remit spontaneously within one month after treatment completion. None of the aforementioned adverse effects constitutes a sufficient reason to discontinue treatment [2]. The commonly used dose is 1 million IU/ml four times per day, as the dose of 3 million IU/ml four times per day has not demonstrated superiority in tumor eradication [7].

The use of topical interferon achieves a higher resolution rate than the use of surgery plus cryotherapy of the base of the lesions, due to the difficulty of completely resecting the lesion without leaving infiltrated borders. In addition, the convenience of topical versus surgical treatment has led to its becoming a widely used treatment nowadays [5]. Not rebiopsy was performed due to the total clinical disappearance of the lesion after topical treatment with INF alfa-2b. After more than 18 months of follow-up, there has been no recurrence. So, it is highly unlikely that remains of the AK exist, since they usually recur with great frequency when that is the case.

Adverse effects due to systemic absorption in the case of the topical administration of the drug are rare, although flu-like and fever conditions are described [7].

Alternatively, topical chemotherapeutic agents, 5-fluoracil, mitomycin C, imiquimod, photodynamic therapy, igenol mebutate, and diclofenac, have been effective [11].

It should be noted that there are no reported cases in the literature of the side effects such as those found in our patient, upper infiltrated upper nasal circular epithelialization and lower nasal congestion, and that these signs decreased drastically within 5 days of cessation of interferon exposure, and the patient was fully recovered from the previously described lesions within one month.

Few cases affecting the conjunctiva have been published [12], only one with conjunctival eyelid margin involvement [2] and none localized on the tarsal conjunctiva.

## 4. Conclusions

Conjunctival AK lesions are scarcely reported in the literature. They are premalignant lesions with the presence of dysplastic keratinocytes. The etiology is due to UV radiation in elderly patients. The treatment of choice is excision with clearance of margins to avoid possible transformation in SCC. Other treatments have been described in the conjunctiva, such as INF alfa-2b, as in this case report. The form of presentation in the tarsal conjunctiva is not described to this day.

## Data Availability

The data used to support the findings of this study are available from the corresponding author upon reasonable request.
